# A Comprehensive Analysis of the Correlations between Resting-State Oscillations in Multiple-Frequency Bands and Big Five Traits

**DOI:** 10.3389/fnhum.2017.00321

**Published:** 2017-06-21

**Authors:** Shigeyuki Ikeda, Hikaru Takeuchi, Yasuyuki Taki, Rui Nouchi, Ryoichi Yokoyama, Yuka Kotozaki, Seishu Nakagawa, Atsushi Sekiguchi, Kunio Iizuka, Yuki Yamamoto, Sugiko Hanawa, Tsuyoshi Araki, Carlos Makoto Miyauchi, Kohei Sakaki, Takayuki Nozawa, Susumu Yokota, Daniele Magistro, Ryuta Kawashima

**Affiliations:** ^1^Department of Ubiquitous Sensing, Institute of Development, Aging and Cancer, Tohoku UniversitySendai, Japan; ^2^Division of Developmental Cognitive Neuroscience, Institute of Development, Aging and Cancer, Tohoku UniversitySendai, Japan; ^3^Division of Medical Neuroimaging Analysis, Department of Community Medical Supports, Tohoku Medical Megabank Organization, Tohoku UniversitySendai, Japan; ^4^Department of Radiology and Nuclear Medicine, Institute of Development, Aging and Cancer, Tohoku UniversitySendai, Japan; ^5^Creative Interdisciplinary Research Division, Frontier Research Institute for Interdisciplinary Science, Tohoku UniversitySendai, Japan; ^6^Human and Social Response Research Division, International Research Institute of Disaster Science, Tohoku UniversitySendai, Japan; ^7^Smart Ageing International Research Center, Institute of Development, Aging and Cancer, Tohoku UniversitySendai, Japan; ^8^School of Medicine, Kobe UniversityKobe, Japan; ^9^Division of Clinical research, Medical-Industry Translational Research Center, Fukushima Medical University School of MedicineFukushima, Japan; ^10^Division of Psychiatry, Tohoku Medical and Pharmaceutical UniversitySendai, Japan; ^11^Department of Functional Brain Science, Institute of Development, Aging and Cancer, Tohoku UniversitySendai, Japan; ^12^Department of Functional Brain Imaging, Institute of Development, Aging and Cancer, Tohoku UniversitySendai, Japan; ^13^Department of Adult Mental Health, National Institute of Mental Health, National Center of Neurology and PsychiatryTokyo, Japan; ^14^Department of Psychiatry, Tohoku University HospitalSendai, Japan; ^15^Graduate School of Arts and Sciences, Department of General Systems Studies, The University of TokyoTokyo, Japan; ^16^Department of Advanced Brain Science, Institute of Development, Aging and Cancer, Tohoku UniversitySendai, Japan; ^17^School of Sport, Exercise, and Health Sciences, Loughborough UniversityLoughborough, United Kingdom; ^18^National Centre for Sport and Exercise Medicine (NCSEM), Loughborough UniversityLoughborough, United Kingdom

**Keywords:** Big Five traits, fALFF, multiple-frequency bands, extraversion, multiple regression analysis

## Abstract

Recently, the association between human personality traits and resting-state brain activity has gained interest in neuroimaging studies. However, it remains unclear if Big Five personality traits are represented in frequency bands (~0.25 Hz) of resting-state functional magnetic resonance imaging (fMRI) activity. Based on earlier neurophysiological studies, we investigated the correlation between the five personality traits assessed by the NEO Five-Factor Inventory (NEO-FFI), and the fractional amplitude of low-frequency fluctuation (fALFF) at four distinct frequency bands (slow-5 (0.01–0.027 Hz), slow-4 (0.027–0.073 Hz), slow-3 (0.073–0.198 Hz) and slow-2 (0.198–0.25 Hz)). We enrolled 835 young subjects and calculated the correlations of resting-state fMRI signals using a multiple regression analysis. We found a significant and consistent correlation between fALFF and the personality trait of extraversion at all frequency bands. Furthermore, significant correlations were detected in distinct brain regions for each frequency band. This finding supports the frequency-specific spatial representations of personality traits as previously suggested. In conclusion, our data highlight an association between human personality traits and fALFF at four distinct frequency bands.

## Introduction

An important challenge in neuroscience is to determine how human personality is represented in the brain. The Big Five model encompasses five personality traits, i.e., neuroticism, extraversion, openness, agreeableness and conscientiousness (Costa and MacCrae, 1992). The development of this model led researchers to ask if the five personality traits correlated with brain activity and which brain areas represented these traits.

Neuroimaging studies have focused on brain activity during resting-state. Previous work revealed that some personality traits correlated with resting-state brain activity. Two studies using positron emission tomography found significant correlations between neuroticism and resting-state activity in the insular cortex and prefrontal regions, and between extraversion and resting-state activity in the orbitofrontal cortex and right putamen (Deckersbach et al., 2006; Kim et al., 2008). Another resting-state study using electroencephalography (EEG) found that both delta and theta activity across all cortical regions significantly correlated with extraversion and conscientiousness (Tran et al., 2006). Recently, functional magnetic resonance imaging (fMRI) was used to investigate correlations between personality traits and resting-state brain activity. Resting-state functional connectivity was associated with each of the five personality traits (Adelstein et al., 2011). Global network measures (i.e., normalized clustering coefficient) from resting-state activity were associated with extraversion (Gao et al., 2013). Neuroticism and extraversion correlated with regional homogeneity of resting-state activity in the left middle frontal gyrus and medial prefrontal cortex, respectively (Wei et al., 2011). Activity in the default mode network (DMN), which is a resting-state network, was associated with extraversion and agreeableness (Sampaio et al., 2014).

To directly determine the amplitude of regional activity during resting-state, a previous study developed an index known as the fractional amplitude of low-frequency fluctuation (fALFF; Zou et al., 2008). fALFF reflects the amplitude of low-frequency (0.01–0.08 Hz) power spectra of hemodynamic signal, and its reliability was previously verified (Zou et al., 2008; Zuo et al., 2010). fALFF was reportedly associated with all of the personality traits except agreeableness (Kunisato et al., 2011).

Previous work defined various frequency classes in wide frequency range (0.02–600 Hz) of electrical activity of the brain (Penttonen and Buzsáki, 2003). Each frequency class is thought to serve different physiological functions (Penttonen and Buzsáki, 2003; Buzsáki and Draguhn, 2004). Fluctuations of hemodynamic activity and electrical activity may have different properties. However, recently, resting-state electrical brain activity was shown to be closely linked to resting-state brain hemodynamics (Matsui et al., 2016). In addition, another resting-state study revealed that patterns of electrical brain activity strongly correlated with that of hemodynamic activity within 0–0.4 Hz frequency band (Ma et al., 2016). The previous findings suggested that the frequency classes defined from electrical activity are beneficial in hemodynamic signal measured with fMRI. Based on the frequency classes, low- and high-frequency fluctuation (0–0.25 Hz) measured with fMRI was decomposed into four distinct frequency bands: (slow-5 (0.01–0.027 Hz), slow-4 (0.027–0.073 Hz), slow-3 (0.073–0.198 Hz) and slow-2 (0.198–0.25 Hz); Zuo et al., 2010).

Only slow-4 and slow-5 bands have been commonly used in resting-state fMRI studies because gray matter-related oscillations during resting-state primarily occur at these two frequency ranges (Zuo et al., 2010). However, resting-state oscillations that included slow-3 showed frequency-dependent spatial structures in the brain (0.01–0.2 Hz; Baria et al., 2011) and limbic resting-state networks (0.01–0.14 Hz; Wu et al., 2008); resting-state networks were consistently present at not only slow-4 and slow-5 but also slow-2 and slow-3 (Gohel and Biswal, 2015); resting-state networks modulated alpha rhythm in different frequency bands such as slow-3 and slow-5 (Zhan et al., 2014); topologies of resting-state networks were different for different frequency bands (including frequencies >slow-4), suggesting that frequency-dependent sub-networks serve different brain functions (Zhang et al., 2015). Therefore, not only slow-4 and slow-5, but also slow-2 and slow-3 should be investigated in resting-state fMRI studies.

fALFF at slow-4 and slow-5 reportedly correlated with extraversion and neuroticism, and brain regions showing significant correlations were different for each frequency band (Wei et al., 2014). It is reasonable to question whether personality traits correlate with fALFF at multiple-frequency bands (including slow-2 and slow-3) and whether spatial distributions of fALFF that significantly correlates with personality traits are different for each band. However, no comprehensive analyses of the associations between the five personality traits and fALFF in four distinct frequency bands have been completed.

In this study, we investigated if fALFF at multiple-frequency bands correlated personality traits. Furthermore, we explored spatial distributions of fALFF that significantly correlated with the personality traits. To this end, personality traits were assessed using the NEO Five-Factor Inventory (NEO-FFI; Costa and MacCrae, 1992), and resting-state activity was measured using fMRI. The fMRI signals were used to calculate fALFF at slow-2–slow-5. Lastly, we performed a multiple regression analysis to determine the correlations between each frequency band and each personality trait.

## Materials and Methods

### Subjects

This study is a part of an ongoing project to investigate the association between brain imaging, cognitive function and aging. We do not describe the psychological tests and MRI scans that were performed during the project because they are out of the scope of this study. Our study included 835 healthy, right-handed subjects (487 males and 348 females, age 20.7 ± 1.8 years). All subjects were university students or postgraduates with normal vision and no history of neurological or psychiatric illness. We used the Edinburgh Handedness Inventory (Oldfield, 1971) to evaluate the subjects’ handedness. We obtained written informed consents from all subjects for their participation in the project. The Ethics Committee of Tohoku University approved all study procedures.

We did not account for the menstrual cycle of female subjects; however, this procedure is similar to most resting-state fMRI studies. Because female subjects were scanned at various stages of the menstrual cycle, the validity of our results may be reduced if the menstrual cycle affects resting-state fMRI measurements.

On the day of psychological tests and MRI scans, all subjects had adequate sleep, ate breakfast, maintained their conditions and were allowed to consume their normal amounts of caffeinated foods and drinks. However, the consumption of alcohol was prohibited beginning on the night before the assessment. The subjects who routinely used certain drugs, such as antipsychotics, were excluded from the project, but the subjects who used daily drugs, such as anti-allergy drugs, were included in the study.

### Image Acquisition

All MRI data were collected using a 3-T Philips Intera Achieva scanner equipped with an 8-channel head coil. The fMRI blood oxygenation level-dependent (BOLD) signal during resting-state was measured with an echo planar imaging sequence (64 × 64 matrix, TR = 2000 ms, TE = 30 ms, flip angle = 70°, FOV = 240 mm, slice thickness = 3.75 mm and 34 transaxial gradient-echo images per volume). A total of 160 functional volumes were acquired while each subject was resting. Based on previously published resting-state fMRI procedures (Greicius et al., 2003; Damoiseaux et al., 2006), the subjects were instructed to keep still with their eyes closed, not to sleep, not to think about anything in particular and remain as motionless as possible.

Using a magnetization-prepared rapid gradient echo sequence, we acquired high-resolution T1-weighted structural images (240 × 240 matrix, TR = 6.5 ms, TE = 3 ms, FOV = 240 mm, slice thickness = 1.0 mm and slices = 162).

In the fMRI measurement, scanner noise was reduced with ear plugs, and pads and Velcro tape were used to limit the subjects’ motion during scanning. The subjects were informed that movement during scanning is unfavorable, and they were instructed to minimize any motions during scanning. If excessive motion was observed, the scan was restarted from the beginning.

### Psychological Measures

To assess the personality traits (i.e., neuroticism, extraversion, openness, agreeableness and conscientiousness) of the subjects, all subjects were asked to complete a 60-item Japanese version (5-point scale) of the NEO-FFI (Costa and MacCrae, 1992; Shimonaka et al., 1999). The five personality traits were previously described as follows (Rosellini and Brown, 2011): (1) neuroticism, the tendency to experience negative emotions and psychological distress in response to stressors; (2) extraversion, the degree of sociability, positive emotionality and general activity; (3) openness, levels of curiosity, independent judgment, and conservativeness; (4) agreeableness, altruistic, sympathetic and cooperative tendencies; and (5) conscientiousness, one’s level of self-control in planning and organization.

To assess intelligence quotient (IQ) of the subjects, the subjects were asked to complete the Tanaka B-type intelligence test (TBIT) type 3B (Tanaka et al., 2003). TBIT is a non-verbal mass intelligence test comprised of subtests that are used for 3rd-year junior high school students and older examinees. In all subtests, the subjects were asked to solve as many problems as possible within the time limit (a few minutes). Our previous work described these subtests in detail (Takeuchi et al., 2013).

### fMRI Data Preprocessing

In this study, we performed the same preprocessing as previously described (Takeuchi et al., 2015). For the preprocessing of imaging data, we used SPM8 (Wellcome Department of Cognitive Neurology, London, UK^1^) implemented in Matlab and SPM8’s extension software Data Processing Assistant for Resting-state fMRI (DPARSF; Chao-Gan and Yu-Feng, 2010).

To avoid treating the skull portions of functional images as the outer edge of the brain parenchyma in the preprocessing procedures, these portions were removed from the first image of a functional image series based on the signal intensity threshold from the spatially smoothed (8 mm FWHM) functional images. The skull-stripped image for each subject was coregistered to a custom made skull-stripped EPI template that we previously created (Takeuchi et al., 2014). For the functional resting-state data of each subject, slice timing correction and realignment were performed using DPARSF. Next, segmentation and normalization were performed using previously described methods (Takeuchi et al., 2014). These methods modified the diffeomorphic anatomical registration through exponentiated lie algebra (DARTEL; Ashburner, 2007) to give functional images with 3.75 × 3.75 × 3.75 mm^3^ voxels. Our previously published Supplemental Methods provide detailed information on the aforementioned procedures (Takeuchi et al., 2015).

To control the effects of head motion and non-neuronal BOLD fluctuations, 27 nuisance covariates included Friston 24 motion parameters and three mean time courses from the voxels within the white matter mask, cerebrospinal fluid (CSF) mask and whole brain mask. According to a previous report, head motion was corrected more effectively using Friston 24 parameters than other movement correction methods, such as the correction for rigid-body using six parameters, derivative 12 parameters and voxel-specific 12 regressors (Yan et al., 2013).

The resultant images were spatially smoothed with an 8 mm FWHM and then masked with a template comprised of 90 regions (without the cerebellar regions) defined by the Anatomical Automatic Labeling (AAL) system (Tzourio-Mazoyer et al., 2002) to exclude the white matter, CSF and cerebellar regions from subsequent analyses. The exclusion of the cerebellar regions is due to technical problems with MRI scanning.

As described previously (Zang et al., 2007; Zou et al., 2008), the fALFF analysis was performed on the aforementioned preprocessed images for each subject. The time series from each voxel (without band-pass filtering) were transformed into the frequency domain with a fast Fourier transform (FFT). The square root was calculated at each frequency of the power spectrum. Based on previous works (Penttonen and Buzsáki, 2003; Buzsáki and Draguhn, 2004; Zuo et al., 2010; Ma et al., 2016; Matsui et al., 2016), the full frequency range (0–0.25 Hz) was divided into four distinct bands: (slow-5 (0.01–0.027 Hz), slow-4 (0.027–0.073 Hz), slow-3 (0.073–0.198 Hz) and slow-2 (0.198–0.25 Hz)). To compute the fALFF of each band, the sum of the amplitude across each band was divided by that of the entire frequency range (i.e., 0–0.25 Hz).

For individual subjects, the fALFF map for each band was transformed to a *Z* score using the global mean of fALFF and standard deviation calculated from the voxels within the 90 AAL regions (Zou et al., 2008).

### Second-Level Analysis

To investigate the correlations between the fALFF values and NEO-FFI scores, we performed a voxel-based multiple regression analysis using SPM8. Nuisance covariates included the IQ score estimated by TBIT, gender, age and total intracranial volume (TIV) to control for their effects because of the potential to influence the association between personality traits and brain function (Hu et al., 2011; Kunisato et al., 2011; Wei et al., 2014). For group voxel-wise statistical testing, a permutation-based non-parametric test (5000 permutations) was performed via the threshold-free cluster enhancement (TFCE) toolbox^2^ (r88; Smith and Nichols, 2009). The resulting TFCE maps were thresholded at *p* < 0.05, a family-wise error (FWE) correction.

## Results

### Behavioral Data

The mean, range and SD of age, IQ, TIV and NEO-FFI score are shown in Table 1. In addition, simple correlations between age, IQ, TIV and NEO-FFI score in each gender group are shown in Supplementary Tables S1, S2.

**Table 1:** The mean, range and standard deviation (SD) of age, intelligence quotient (IQ), TIV and NEO Five-Factor Inventory (NEO-FFI) score in male group, female group and mixed gender group.

	All			Male			Female		
	Mean	range	SD	Mean	range	SD	Mean	range	SD
Age	20.7	18–27	1.8	20.7	18–27	1.9	20.6	18–27	1.7
IQ	112.3	78–147	11.7	114.2	79–147	11.6	109.6	78–143	11.3
TIV [cm^3^]	1539	1215–2018	141	1616	1306–2018	115	1431	1215–1724	94
N	28.0	3–48	8.4	27.5	4–47	8.4	28.7	3–48	8.3
E	26.4	7–47	6.8	26.0	7–47	7.1	27.0	7–39	6.4
O	29.6	11–47	5.9	29.1	13–47	5.8	30.2	11–45	5.9
A	30.5	5–45	6.1	29.4	5–45	6.0	32.1	11–43	5.8
C	25.4	3–42	6.9	25.0	3–41	7.0	26.0	7–42	6.7

*TIV, total intracranial volume; N, neuroticism; E, extraversion; O, openness; A, agreeableness; C, conscientiousness*.

### Positive Correlations between fALFF at Multiple-Frequency Bands and Extraversion

**Figure 1:**
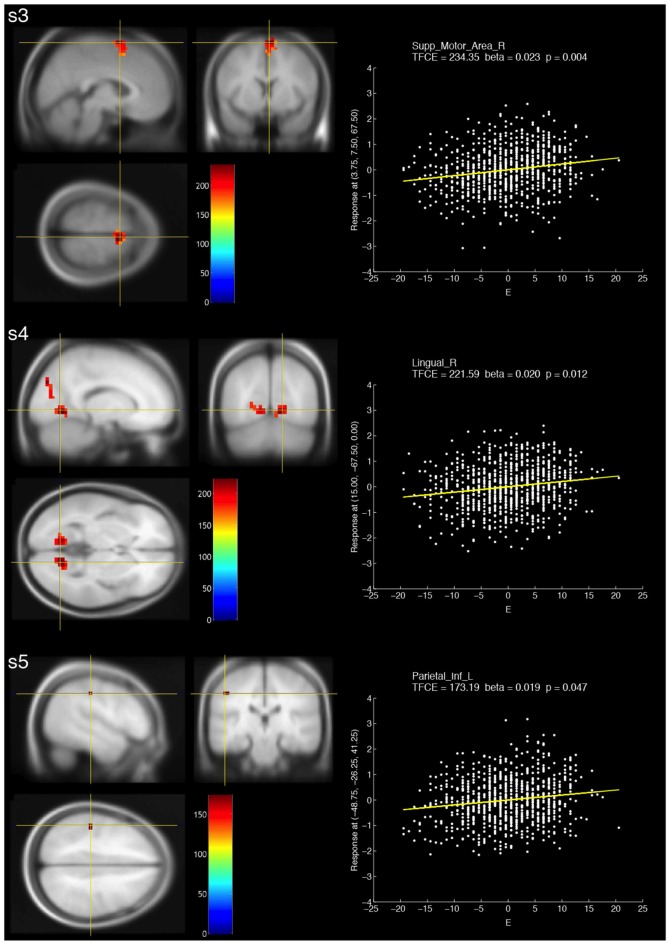
Brain areas that showed significant positive correlations with fractional amplitude of low-frequency fluctuation (fALFF) at three different frequency bands (s3: slow-3, s4: slow-4 and s5: slow-5) and extraversion scores (E). The resulting threshold-free cluster enhancement (TFCE) maps were thresholded with a voxel-wise significance of a family-wise error (FWE)-corrected *p* < 0.05 and overlaid on the avg305T1 template using SPM8. The color bar indicates the TFCE magnitude. The peak voxels for the three bands are marked by the yellow cross-hairs. Each scatter plot shows the relationship between the E score (minus the mean value) and fALFF at the peak voxel. The nuisance covariates were regressed out at the peak voxel. Each yellow line within the scatter plot shows the fALFF predicted from the E score.

The resulting TFCE maps with a voxel-wise threshold (FWE-corrected *p* < 0.05) revealed significant positive correlations between fALFF at three different frequency bands (slow-3, slow-4 and slow-5) and extraversion scores (Figure 1, Table 2). For slow-3, a large cluster showing significant positive correlations was observed in the bilateral supplementary motor and right medial superior frontal areas. For slow-4, the significant positive correlations formed two large clusters, including the bilateral calcarine, bilateral lingual, bilateral cuneus and left superior occipital areas. For slow-5, a small cluster was observed in the left postcentral and left inferior parietal areas.

**Table 2:** Brain areas in which fractional amplitude of low-frequency fluctuation (fALFF) at each frequency band significantly correlated with NEO-FFI scores.

		Cluster size, AAL areas and percentages for entire voxels	Peak coordinates			AAL areas	TFCE	*P*_FWE_
			*x*	*y*	*z*			
S2	E (< 0)	1	0	−86.25	33.75	QG	161.07	0.046
	C (> 0)	6	−60.00	0	33.75	FAG	170.56	0.032
		FAG 33.33%
		PAG 66.67%
S3	E (> 0)	54	3.75	7.50	67.50	SMAD	234.35	0.004
		SMAG 24.07%
		SMAD 74.07%
		FMD 1.85%
S4	E (> 0)	Cluster 1: 35	15.00	−67.50	0	LINGD	221.59	0.012
		V1D 14.29%
		LINGD 85.71%
		Cluster 2: 97	0	−86.25	33.75	QG	217.28	0.013
		V1G 19.59%
		V1D 4.12%
		QG 34.02%
		QD 18.56%
		LINGG 22.68%
		O1G 1.03%
	E (< 0)	Cluster 1: 5	18.75	56.25	3.75	F1D	173.52	0.042
		F1D 40.00%
		FMD 60.00%
		Cluster 2: 2	18.75	56.25	18.75	F1D	171.21	0.045
		F1D 100.00%
		Cluster 3: 2	22.50	60.00	33.75	F1D	181.75	0.03
		F1D 100.00%
S5	E (> 0)	3	−48.75	−26.25	41.25	P2G	173.19	0.047
		PAG 33.33%
		P2G 66.67%

*S2: slow-2; S3: slow-3; S4: slow-4; S5: slow-5; E: extraversion; C: conscientiousness; L: left; R: right; FAG: Precentral_L; F1D: Frontal_Sup_R; SMAG: Supp_Motor_Area_L; SMAD: Supp_Motor_Area_R; FMD: Frontal_Sup_Medial_R; V1G: Calcarine_L; V1D: Calcarine_R; QG: Cuneus_L; QD: Cuneus_R; LINGG: Lingual_L; LINGD: Lingual_R; O1G: Occipital_Sup_L; PAG: Postcentral_L; P2G: Parietal_Inf_L (for details on the abbreviations of the anatomical areas, see Tzourio-Mazoyer et al., 2002)*.

### Negative Correlations between fALFF at Multiple-Frequency Bands and Extraversion

**Figure 2:**
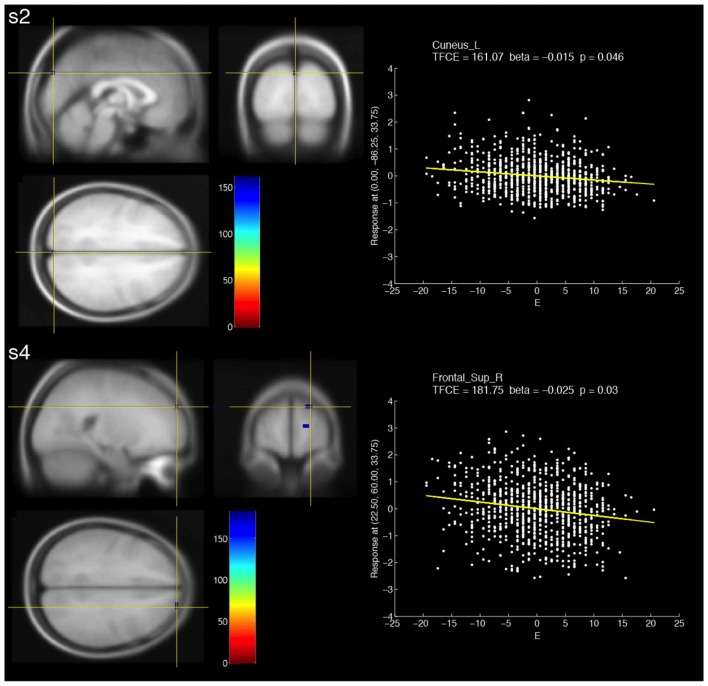
Brain areas that showed significant negative correlations with fALFF at two different frequency bands (s2: slow-2, s4: slow-4) and extraversion scores (E). The resulting TFCE maps were thresholded with a voxel-wise significance of a FWE-corrected *p* < 0.05 and overlaid on the avg305T1 template using SPM8. The color bar indicates the TFCE magnitude. The peak voxels for the two bands are marked by the yellow cross-hairs. Each scatter plot shows the relationship between the E score (minus the mean value) and fALFF at the peak voxel. The nuisance covariates were regressed out at the peak voxel. Each yellow line within the scatter plot shows the fALFF predicted from the E score.

When compared with the positive correlations, smaller clusters were observed for negative correlations (Figure 2, Table 2). For slow-2, a voxel in the left cuneus showed a significant negative correlation. For slow-4, three small clusters that showed significant negative correlations were observed in the right superior frontal and right medial superior frontal areas.

### Correlations between fALFF and Personality Scores Other than Extraversion

For slow-2, a cluster that showed a significant positive correlation for conscientiousness (Table 2) was observed in the left precentral and left postcentral areas. No other significant correlations were found.

## Discussion

Our work is the first comprehensive study to investigate the correlations between fALFF at four distinct frequency bands (slow-2–slow-5) and the five personality traits assessed using the NEO-FFI. Our hypothesis was that personality traits correlate with fALFF at multiple-frequency bands, including slow-2 and slow-3. Furthermore, we explored spatial distributions of the significant correlations. The results obtained showed that significant correlations between fALFF and extraversion were consistently present at all frequency bands, thus supporting our hypothesis. Furthermore, most of the significant correlations were distributed over distinct brain regions for each frequency band, supporting frequency-specific spatial representations of personality traits as previously suggested (Wei et al., 2014). Interestingly, we found no other significant correlations, except for a correlation between conscientiousness and fALFF at slow-2. Our results shed light on the association between human personality traits and fALFF at four distinct frequency bands.

For extraversion, significant correlations were present at all frequency bands. Three relatively large clusters were found at slow-3 and slow-4 bands, while small spatial extent was found at slow-2 and slow-5. Except for extraversion and conscientiousness, the other traits did not show significant correlations. These results suggest the robustness of extraversion representations in fALFF. According to previous findings, extraversion is considered the most stable core trait (Lei et al., 2015), and correlations between extraversion and fALFF (0.01–0.08 Hz, slow-4 and slow-5) formed larger clusters relative to the other personality traits (Kunisato et al., 2011; Wei et al., 2014). Our results support the previous findings in terms of the robustness of extraversion. Furthermore, the significant correlations of slow-2 and slow-3 appear to be novel findings.

For positive correlations between slow-5 and extraversion, we found a small cluster formed by voxels within the left postcentral and inferior parietal regions. The results were partially consistent with the previous findings, which showed significant positive correlations between extraversion and fALFF at slow-5 in precuneus, which is a part of the parietal lobe (Wei et al., 2014). Furthermore, extraversion was found to be associated with the Hurst exponent (H), which is an indicator calculated from power spectra (0.016–0.063 Hz), in a right-lateralized frontoparietal network (Lei et al., 2013). These evidences suggest that the parietal lobe is linked to extraversion.

For positive correlations between slow-4 and extraversion, we found two large clusters formed by voxels within the occipital lobe, such as the bilateral calcarine, cuneus and lingual areas. The results were inconsistent with previous findings that showed significant correlations between extraversion and fALFF at slow-4 in hippocampus (Wei et al., 2014), and between extraversion and fALFF (0.01–0.08 Hz), which encompasses slow-4, in the striatum, bilateral precuneus and right superior frontal gyrus (Kunisato et al., 2011). On the other hand, although H and fALFF may have different properties, extraversion was found to be associated with H in the occipital lobe (Lei et al., 2013). In terms of the cuneus in which significant voxels were observed, some previous studies suggested the importance of the cuneus in extraversion. For example, the cuneus was activated by watching risk-taking actions, and the degree of feeling “risky” and “anxiety” about risk-taking actions negatively correlated with extraversion (Tamura et al., 2012). Further, high extraversion was associated with greater activity in the left cuneus during rest (Ruffle et al., 2015). Lastly, extraversion positively correlated with short-range functional connectivity density in the left cuneus (Pang et al., 2015).

For negative correlations between slow-4 and extraversion, we found some small clusters formed by voxels within the right superior frontal region. The results were inconsistent with the previous findings (Wei et al., 2014). On the other hand, fALFF (0.01–0.08 Hz) was found to positively correlate with extraversion in the right superior frontal gyrus (Kunisato et al., 2011). Note that the direction of the correlation was inconsistent with that of our results. A right-lateralized frontoparietal network was associated with extraversion (Lei et al., 2013). These evidences suggest the link between the right frontal region and extraversion.

For positive correlations between slow-3 and extraversion, we found a large cluster in the bilateral supplementary motor areas (SMA) and right medial superior frontal area; the peak voxel within this cluster showed the greatest TFCE across all frequency bands. Regional homogeneity, which is an indicator different from fALFF, in the medial prefrontal cortex was found to correlate with extraversion (Wei et al., 2011). SMA is one of the functional subdivisions in Brodmann area (BA) 6 (Hanakawa et al., 2002). Previous work showed that fALFF (0.01–0.08 Hz), which is different from slow-3 (0.073–0.198 Hz), in BA6 positively correlated with extraversion (Kunisato et al., 2011). The SMA was activated by watching “risk-taking” actions, and risk-taking actions were associated with extraversion (Tamura et al., 2012). The SMA is associated with performing voluntary actions rather than involuntary actions (Thaler et al., 1995). High extraversion is directly linked to traits such as talkativeness and aggressiveness. These traits may be associated with voluntary actions controlled by the SMA. Therefore, our results are reasonable based on the previous findings.

For a negative correlation between slow-2 and extraversion, we observed only one voxel in the left cuneus. It is interesting that fALFF at slow-2 and slow-4 showed significant correlations in the same region (i.e., the left cuneus), and that the directions of the correlations were opposite. This fact suggests that representations of extraversion in the left cuneus are different for each band.

We found no significant correlations for the other personality traits, except for extraversion and conscientiousness. fALFF (0.01–0.08 Hz) reportedly correlates with not only extraversion and conscientiousness but other traits (i.e., neuroticism and openness; Kunisato et al., 2011). The inconsistency between our results and previous findings may be explained by the differences in sample sizes and analysis methods (e.g., motion correction, signal regression and nuisance covariates). This study included 835 subjects, which is a relatively large sample size in the neuroimaging field. For preprocessing, we used the Friston 24 model for motion correction and performed a global signal regression. A previous study showed the benefit of combining the Friston 24 model with global signal regression in fALFF (see Figure S10 in Yan et al., 2013). In the second-level analysis, IQ, gender, age and TIV were used as nuisance covariates. The use of these covariates was based on previous studies (Hu et al., 2011; Kunisato et al., 2011; Wei et al., 2014). Thus, we believe that our results obtained by the aforementioned analyses are reliable.

For slow-2 and slow-3, we found the significant correlations in extraversion and conscientiousness; in addition, the positive correlations between extraversion and slow-3 formed a relatively large cluster. Resting-state BOLD activity at slow-2 and slow-3 bands is mainly distributed over white matter (Zuo et al., 2010), and the BOLD activity at slow-2 and slow-3 involved respiratory and aliased cardiac signals (Cordes et al., 2001). Cardiac signals are known to correlate with the personality (Koelsch et al., 2012). Previous resting-state fMRI studies using fALFF did not use the signals at slow-2 and slow-3 to avoid the influence of such physiological noise (Han et al., 2010; Kunisato et al., 2011; Wei et al., 2014; Yu et al., 2014). Because physiological signals (i.e., respiratory and cardiac signals) were not measured in the present study, the influence of such signals on BOLD fMRI data could not be quantitatively assessed. On the other hand, reportedly, the global signal regression approach used in the present study contributes to the reduction in physiological noise such as respiratory and cardiac signals because much of the global brain signal is due to the physiological processes (Birn et al., 2006; Chang and Glover, 2009). Furthermore, even though hemodynamic response was attenuated in higher frequency bands (slow-2 and slow-3), the BOLD signal formed still resting-state networks (Gohel and Biswal, 2015). This fact suggests that the BOLD fluctuations in slow-2 and slow-3 have a significant presence of neuronal fluctuations, and supports our results. Therefore, it is reasonable to use fALFF in slow-2 and slow-3 frequency bands in resting-state fMRI research.

For physiological meaning of the fALFF bands, reportedly different bands may play different roles in resting-state BOLD activity (Zhan et al., 2014); in particular, fMRI data at the slow-3 and slow-5 bands correlated with the EEG alpha power, and spatial patterns of the correlations showed different brain networks for different bands; the slow-2 and slow-4 bands rarely correlated with the EEG alpha power. Therefore, the alpha rhythm was influenced by different networks in different bands (i.e., slow-3 and slow-5), while slow-2 and slow-4 may have other physiological meanings. Another previous study reported that fMRI signals in the 0.15–0.25 Hz (including slow-2) and 0.03–0.08 Hz (including slow-4) frequency bands, showed different topologies of resting-state networks (Zhang et al., 2015). Together, the frequency-specific characteristics of the resting-state brain may link to the frequency-specific spatial representations of personality traits, which were observed in the present study.

Many of the correlations observed in this study were only marginally significant. In neuroscience, the effects explored in experiments reportedly tend to be smaller and more subtle (Button et al., 2013). Therefore, in our experiment, the true effect size of association between human personality traits and fALFF may be small. To detect small effects, a large sample size is needed (Button et al., 2013). Our study included 835 subjects, which is a relatively large sample size for neuroimaging studies. We believe that the large sample size contributed to the detection of the association between personality traits and fALFF.

Although we observed the consistent correlations between all frequency bands and extraversion, extraversion is likely to correlate with physiological noises which may affect fALFF. For example, extraverts may have difficulties in keeping quiet in the fMRI scanner, so that this may cause severe movement artifacts, which may persist even after motion artifact correction. To assess an association between movement artifacts and extraversion, we calculated a correlation between mean framewise displacement (Power et al., 2012) and extraversion (*r* = 0.042, *p* = 0.228). The correlation was not significant, so that extraversion should exert little influence on movement artifacts.

The present study has important limitations:

1. In the second-level analysis, 40 multiple comparisons were performed (i.e., 5 personality traits × 4 frequency bands × 2 directions). The obtained results were likely to be due to spurious false positives. To confirm the possibility, we reanalyzed the fMRI data also without using the global signal regression. If our results were derived from true effects, results of this reanalysis were expected to show correlation patterns similar to that of our results. As a result, we observed similar correlation patterns, i.e., extraversion consistently correlated with fALFF at all frequency bands, while no other traits correlated with fALFF (Supplementary Table S3). Therefore, the obtained results in the present study must be due to true effects.
2. Activities of whole cerebellar regions could not be fully measured due to technical problems with MRI scanning. Reportedly, fALFF (0.01–0.08 Hz) was found to correlate with conscientiousness in cerebellar regions (Kunisato et al., 2011). Therefore, further investigation of associations between fALFF at multiple-frequency bands and personality traits in cerebellar regions should provide more information about frequency-specific spatial representations of personality traits.
3. In the present study, the upper limit of the slow-2 band (0.198–0.25 Hz) was constrained by the fMRI repetition time to acquire BOLD signals (TR = 2000 ms); however, the true slow-2 band was defined as 0.198–0.5 Hz (Penttonen and Buzsáki, 2003). The upper limit may explain the small effects in slow-2.
4. The present study could not provide information about temporal reliability of the observed correlations between fALFF and personality traits. However, fALFF has been shown to exhibit high test-retest reliability (Zuo et al., 2010). This fact supports the temporal reliability of the correlations observed in the present study.

In conclusion, we investigated if fALFF at multiple-frequency bands (slow-2–slow-5) correlated with personality traits assessed using the NEO-FFI. We also investigated if spatial distributions of fALFF that significantly correlated with personality traits were different for each band. The significant correlations between fALFF and extraversion were present at all frequency bands, suggesting the importance of high frequency bands (>slow-4) in traits represented in fALFF. Furthermore, most of voxels showing significant correlations were observed in different areas for each frequency band. This finding supports the previous suggestion of frequency-specific spatial representations of personality traits. Lastly, our study provided a comprehensive analysis of the association between four distinct frequency bands and the five personality traits of the Big Five Model.

## Author Contributions

HT designed and developed the study protocol. All authors collected data. SI conducted the study and analyzed the data; prepared the manuscript with the other authors. All authors read and approved the final manuscript.

## Conflict of Interest Statement

The authors declare that the research was conducted in the absence of any commercial or financial relationships that could be construed as a potential conflict of interest.

## Footnotes

^1^http://www.fil.ion.ucl.ac.uk/spm/

^2^http://dbm.neuro.uni-jena.de/tfce/
