# Supplementary material for: A Comprehensive Analysis of the Correlations between Resting-State Oscillations in Multiple-Frequency Bands and Big Five Traits
Source: Front Hum Neurosci. 2017 Jun 21;11:321. doi: 10.3389/fnhum.2017.00321 (PMC5478695; doi:10.3389/fnhum.2017.00321)
Supplement: Supplementary file 1 [file Table_1.docx]

**Table S1** Simple correlations between age, IQ, total intracranial volume (TIV), and the five personality traits (N, E, O, A, and C) in the male subjects.

|  | Age | IQ | TIV | N | E | O | A | C |
| --- | --- | --- | --- | --- | --- | --- | --- | --- |
| Age | - | 0.014 0.758 | 0.084 0.064 | **-0.246 3.61×10^-8^** | **0.151 0.001** | 0.029 0.518 | **0.135 0.003** | **0.168 1.96×10^-4^** |
| IQ |  | - | **0.13 0.004** | -0.034 0.461 | 0.042 0.356 | 0.084 0.064 | 0.042 0.351 | 0.013 0.768 |
| TIV |  |  | - | -0.005 0.919 | -0.052 0.255 | -0.025 0.587 | **-0.103 0.023** | 0.002 0.96 |
| N |  |  |  | - | **-0.354 7.53×10^-16^** | 0.011 0.805 | **-0.275 6.87×10^-10^** | **-0.314 1.26×10^-12^** |
| E |  |  |  |  | - | **0.132 0.004** | **0.37 2.75×10^-17^** | **0.276 5.95×10^-10^** |
| O |  |  |  |  |  | - | 0.069 0.126 | -0.019 0.677 |
| A |  |  |  |  |  |  | - | **0.156 0.001** |
| C |  |  |  |  |  |  |  | - |

In each cell, upper values indicate correlation values and lower values indicate p values. Bold font indicates correlations that were statistical significant (< 0.05).
