# Supplementary material for: A Comprehensive Analysis of the Correlations between Resting-State Oscillations in Multiple-Frequency Bands and Big Five Traits
Source: Front Hum Neurosci. 2017 Jun 21;11:321. doi: 10.3389/fnhum.2017.00321 (PMC5478695; doi:10.3389/fnhum.2017.00321)
Supplement: Supplementary file 2 [file Table_2.docx]

**Table S2** Simple correlations between age, IQ, total intracranial volume (TIV), and the five personality traits (N, E, O, A, and C) in the female subjects.

|  | Age | IQ | TIV | N | E | O | A | C |
| --- | --- | --- | --- | --- | --- | --- | --- | --- |
| Age | - | 0.093 0.082 | 0.058 0.278 | **-0.129 0.016** | **0.123 0.022** | 0.026 0.635 | **0.146 0.006** | 0.031 0.568 |
| IQ |  | - | **0.15 0.005** | 0.003 0.952 | 0.013 0.803 | 0.093 0.083 | -0.045 0.403 | -0.043 0.426 |
| TIV |  |  | - | 0.001 0.992 | 0.012 0.822 | -0.025 0.643 | 0.056 0.294 | -0.006 0.912 |
| N |  |  |  | - | **-0.294 2.35×10^-8^** | -0.056 0.299 | **-0.334 1.67×10^-10^** | **-0.341 6.55×10^-11^** |
| E |  |  |  |  | - | **0.153 0.004** | **0.392 3.40×10^-14^** | **0.214 5.61×10^-5^** |
| O |  |  |  |  |  | - | 0.098 0.069 | -0.043 0.425 |
| A |  |  |  |  |  |  | - | **0.169 0.002** |
| C |  |  |  |  |  |  |  | - |

In each cell, upper values indicate correlation values and lower values indicate p values. Bold font indicates correlations that were statistical significant (< 0.05).
