# Supplementary material for: A Comprehensive Analysis of the Correlations between Resting-State Oscillations in Multiple-Frequency Bands and Big Five Traits
Source: Front Hum Neurosci. 2017 Jun 21;11:321. doi: 10.3389/fnhum.2017.00321 (PMC5478695; doi:10.3389/fnhum.2017.00321)
Supplement: Supplementary file 3 [file Table_3.docx]

**Table S3** Brain areas in which fALFF at each frequency band significantly correlated with NEO-FFI scores. Note that fALFF was calculated from fMRI data without the global signal regression.

|  |  | Cluster size, AAL areas, and percentages for entire voxels | \| Peak coordinates \| \| \| \| --- \| --- \| --- \| \| x \| y \| z \| | | | AAL areas | TFCE | P_FWE_ |
| --- | --- | --- | --- | --- | --- | --- | --- | --- | --- | --- | --- | --- | --- | --- |
| S2 | E (> 0) | Cluster 1: 8  Cingulum_Post_R, 25.00%  Lingual_R, 12.50%  Precuneus_R, 62.50% | 7.50 | -52.50 | 7.50 | Precuneus_R | 179.45 | 0.030 |
|  |  | Cluster 2: 98  Calcarine_L, 1.02%  Cuneus_L, 26.53%  Cuneus_R, 5.10%  Occipital_Sup_L, 1.02%  Precuneus_L, 39.80%  Precuneus_R, 26.53% | -3.75 | -75.00 | 33.75 | Cuneus_L | 248.23 | 0.004 |
|  |  | Cluster 3: 3  Precuneus_R, 100.00% | 11.25 | -78.75 | 48.75 | Precuneus_R | 171.93 | 0.040 |
|  | E (< 0) | Cluster 1: 200  Rolandic_Oper_L, 3.00%  Insula_L, 8.00%  Putamen_L, 7.50%  Pallidum_L, 0.50%  Heschl_L, 5.50%  Temporal_Sup_L, 31.00%  Temporal_Pole_Sup_L, 1.00%  Temporal_Mid_L, 43.50% | -60.00 | -26.25 | 3.75 | Temporal_Sup_L | 323.06 | 0.000 |
|  |  | Cluster 2: 7  Occipital_Inf_L, 100.00% | -45.00 | -78.75 | -11.25 | Occipital_Inf_L | 184.52 | 0.032 |
|  |  | Cluster 3: 1  Temporal_Sup_L, 100.00% | -48.75 | -3.75 | -15.00 | Temporal_Sup_L | 170.15 | 0.047 |
|  |  | Cluster 4: 3  Insula_R, 100.00% | 37.50 | -11.25 | 7.50 | Insula_R | 174.29 | 0.042 |
|  |  | Cluster 5: 12  Precentral_L, 16.67%  Postcentral_L, 83.33% | -56.25 | -3.75 | 22.50 | Postcentral_L | 182.97 | 0.033 |
|  |  | Cluster 6: 3  Postcentral_L, 100.00% | -41.25 | -30.00 | 45.00 | Postcentral_L | 178.80 | 0.037 |
|  |  | Cluster 7: 3  Parietal_Sup_L, 100.00% | -30.00 | -45.00 | 71.25 | Parietal_Sup_L | 176.70 | 0.038 |
| S3 | E (> 0) | Cluster 1: 7  Frontal_Sup_L, 14.29%  Frontal_Sup_Medial_L, 85.71% | -3.75 | 37.50 | 45.00 | Frontal_Sup_Medial_L | 196.76 | 0.035 |
|  |  | Cluster 2: 16  Supp_Motor_Area_R, 93.75%  Frontal_Sup_Medial_R, 6.25% | 11.25 | 18.75 | 60.00 | Supp_Motor_Area_R | 232.58 | 0.013 |
|  | E (< 0) | Cluster 1: 32  Lingual_R, 96.88%  Fusiform_R, FUSID, 3.12% | 15.00 | -60.00 | -3.75 | Lingual_R | 210.89 | 0.022 |
|  |  | Cluster 2: 24  Rolandic_Oper_R, 29.17%  Heschl_R, 4.17%  Temporal_Sup_R, 62.50%  Temporal_Pole_Sup_R, 4.17% | 63.75 | 0.00 | 7.50 | Rolandic_Oper_R | 208.29 | 0.024 |
|  |  | Cluster 3: 4  Temporal_Sup_L, 50.00%  Temporal_Mid_L, 50.00% | -52.50 | -18.75 | 0.00 | Temporal_Sup_L | 192.43 | 0.037 |
|  |  | Cluster 4: 4  Postcentral_L, 100.00% | -48.75 | -7.50 | 15.00 | Postcentral_L | 185.98 | 0.044 |
|  |  | Cluster 5: 11  Cuneus_L, 36.36%  Occipital_Sup_L, 63.64% | -22.50 | -82.50 | 33.75 | Occipital_Sup_L | 188.90 | 0.041 |
|  |  | Cluster 6: 7  Cuneus_R, 100.00% | 11.25 | -82.50 | 33.75 | Cuneus_R | 189.73 | 0.040 |
|  |  | Cluster 7: 8  Postcentral_L, 50.00%  Parietal_Sup_L, 25.00%  Precuneus_L, 25.00% | -18.75 | -41.25 | 67.50 | Postcentral_L | 196.84 | 0.033 |
| S4 | E (> 0) | Cluster 1: 591  Calcarine_L, 16.24%  Calcarine_R, 15.91%  Cuneus_L, 9.64%  Cuneus_R, 9.81%  Lingual_L, 19.97%  Lingual_R, 18.95%  Occipital_Sup_L, 1.35%  Occipital_Sup_R, 1.86%  Occipital_Mid_L, 0.68%  Occipital_Inf_L, 2.37%  Fusiform_L, 0.68%  Fusiform_R, 2.54% | 15.00 | -67.50 | -3.75 | Lingual_R | 394.80 | 0.000 |
|  |  | Cluster 2: 162  Rolandic_Oper_L, 6.17%  Insula_L, 4.32%  Postcentral_L, 8.02%  Heschl_L, 8.64%  Temporal_Sup_L, 50.00%  Temporal_Mid_L, 22.84% | -33.75 | -30.00 | 7.50 | Heschl_L | 267.68 | 0.007 |
|  | E (< 0) | Cluster 1: 596  Frontal_Sup_L, 16.61%  Frontal_Sup_R, 11.91%  Frontal_Sup_Orb_L, 3.52%  Frontal_Sup_Orb_R, 1.68%  Frontal_Mid_L, 3.36%  Frontal_Mid_R, 1.34%  Frontal_Mid_Orb_L, 7.38%  Frontal_Mid_Orb_R, 7.38%  Frontal_Inf_Tri_L, 1.68%  Frontal_Inf_Orb_L, 1.51%  Frontal_Inf_Orb_R, 4.53%  Frontal_Sup_Medial_L, 8.89%  Frontal_Sup_Medial_R, 21.14%  Cingulum_Ant_L, 0.84%  Cingulum_Ant_R, 8.22% | 18.75 | 63.75 | 11.25 | Frontal_Sup_R | 376.39 | 0.001 |
|  |  | Cluster 2: 1  Frontal_Med_Orb_L, 100.00% | -3.75 | 67.50 | -15.00 | Frontal_Med_Orb_L | 186.18 | 0.049 |
|  |  | Cluster 3: 1  Frontal_Inf_Tri_R, 100.00% | 45.00 | 30.00 | 0.00 | Frontal_Inf_Tri_R | 186.74 | 0.048 |
|  |  | Cluster 4: 11  Frontal_Sup_L, 90.91%  Frontal_Sup_Medial_L, 9.09% | -11.25 | 33.75 | 37.50 | Frontal_Sup_L | 204.91 | 0.028 |
|  |  | Cluster 5: 6  Frontal_Mid_L, 100.00% | -30.00 | 26.25 | 60.00 | Frontal_Mid_L | 205.81 | 0.027 |
|  |  | Cluster 6: 9  Frontal_Sup_R, 11.11%  Supp_Motor_Area_R, 88.89% | 15.00 | 15.00 | 63.75 | Supp_Motor_Area_R | 204.13 | 0.029 |
| S5 | E (> 0) | Cluster 1: 1  Temporal_Sup_R, 100.00% | 60.00 | -11.25 | -7.50 | Temporal_Sup_R | 191.43 | 0.049 |
|  |  | Cluster 2: 31  Temporal_Sup_L, 32.26%  Temporal_Mid_L, 67.74% | -48.75 | -30.00 | 0.00 | Temporal_Mid_L | 271.30 | 0.007 |
|  |  | Cluster 3: 4  Temporal_Sup_R, 100.00% | 45.00 | -26.25 | 0.00 | Temporal_Sup_R | 198.00 | 0.042 |

S2: slow-2; S3: slow-3; S4: slow-4; S5: slow-5; E: extraversion; L: left; R: right.
